# What remains of the evidence for auxin feedback on PIN polarity patterns?

**DOI:** 10.1093/plphys/kiab118

**Published:** 2021-03-24

**Authors:** Kirsten H ten Tusscher

**Affiliations:** Department of Theoretical Biology, Faculty of Sciences, Utrecht University, Padualaan 8, 3584 CH, Utrecht, The Netherlands

## Abstract

In light of recent findings, the feedback between auxin and PIN that plays a major role in models for self-organized auxin patterning requires revisiting.

Dear Editor,

Auxin patterns play critical roles in a wide range of processes in plant development and adaptation, with auxin maxima indicating the location of future lateral root forming competent sites (De Smet et al., 2007) and auxin asymmetries guiding various plant tropic responses (Galvan-Ampudia et al., 2013) to mention two examples.

While auxin production, degradation, and import into cells play important modulatory roles in plant auxin patterning (Ljung et al., 2005; Band et al., 2014; Di Mambro et al, 2017), a major role is ascribed to the PIN-FORMED auxin exporters (PINs) (Paponov et al., 2005). PIN proteins, due to their polar plasma membrane distribution, guide directional polar auxin transport (PAT) that strongly dictates tissue scale auxin patterns. Indeed, dynamic changes in PIN polarity patterns underlie initiation and termination of tropic responses (Rakusova et al., 2015) and formation of new organs and body axes during, for example, shoot phyllotaxis and lateral root formation (Du and Scheres, 2018). Major questions in the field thus revolve around which mechanisms govern PIN polarity patterns and dynamic changes therein.

Of particular interest is the question of whether and how auxin itself, whose pattern is strongly determined by PIN-mediated PAT, may affect PIN polarity patterns. In mathematical terms, a (non-linear) positive feedback between auxin and its transporter can give rise to the so-called bistability, enabling a stimulus to result in a switch in auxin levels and polarity level or orientation that is subsequently stably maintained (Figure 1A). Thus, a feedback loop between auxin and PINs would possibly enable self-organized auxin patterning and auxin-stimulus-dependent changes therein (Van Berkel et al., 2013). The question of self-organized auxin patterning has a long history of inspiring mathematical and computational modeling. Modeling attempts predominantly focused on explaining the formation of auxin maxima guiding phyllotactic leaf placement (Figure 1B), the auxin streams pre-defining vascular patterns (Figure 1C), and more recently their combination. In the case of phyllotaxis, the so-called “up-the-gradient” type feedback mechanism with cells polarizing their PIN proteins toward neighboring cells with highest auxin levels, can amplify small initial differences into regularly spaced auxin maxima (Smith et al., 2006; Jönsson et al., 2006; Newell et al., 2007). Put simply, by enhancing PIN levels on membranes facing the neighbor cell with the highest auxin levels, even more auxin is directed toward this cell, causing auxin maxima and PIN-mediated auxin streams toward them to first be amplified and then maintained. For venation, the canalization hypothesis put forward by Sachs (1969), suggesting that cells enhance their auxin efflux across the cellular membrane domains experiencing the largest auxin efflux, can form auxin flux canals through “with-the-flux” feedback (Mitchison 1980; Feugier et al., 2005; Fujita and Mochizuki, 2006; Stoma et al., 2008; Alim and Frey, 2010). Here, by enhancing PIN levels on membranes experiencing the highest efflux rates, this efflux is first further enhanced and then maintained.

**Figure 1:**
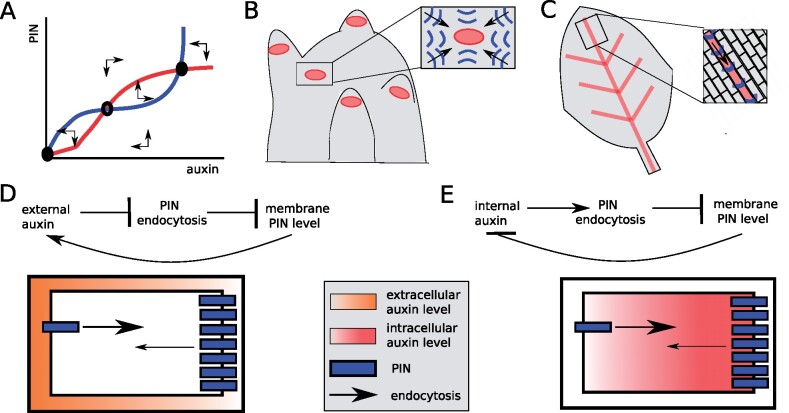
Auxin feedback on PIN levels and polarity as a driver of plant auxin patterning. A, Phase plane depiction of a two-variable auxin PIN model (for more details see e.g. Van Berkel et al., 2013). A, Positive nonlinear feedback from auxin on PIN levels results in bistability, with a stable low auxin-low PIN equilibrium (lower left black dot) separated from a stable high auxin-high PIN equilibrium (upper right black dot) by an instable intermediate auxin-intermediate PIN equilibrium (middle dot). B, In models, up-the-gradient polarization of PINs results in the regularly spaced auxin maxima observed during phyllotaxis. C, In models, with-the-flux polarization of PINs results in the formation of auxin canals observed during venation. D, Paciorek et al. (2005) reported a negative effect of auxin on PIN endocytosis. If the auxin effect is exerted by external, apoplastic auxin this results in a positive feedback and PIN polarization. E, Narasimhan et al. (2021) instead reported a positive, PIN2-specific effect on endocytosis. Here, to obtain a positive feedback between auxin and PIN that results in PIN polarization, the auxin effect would need to be exerted by an internal auxin.

While early models were largely phenomenological, recent models seek to explore the potential mechanistic basis underlying auxin (flux) sensing as well as mechanisms enabling both up-the-gradient and with-the-flux type patterning. Models have proposed intra-apoplast auxin gradient sensing (Wabnik et al., 2010) or mechanical sensing (Heisler et al., 2010) as an alternative to the sensing of neighboring cells auxin levels for which molecular mechanisms are hard to conceive. Additionally, models have proposed tallying mechanisms through which cells, either directly via the auxin transporters or via in parallel produced tally molecules, could measure auxin influx and efflux (Cieslak et al., 2015). Through letting extracellular auxin levels modulate the extent to which auxin efflux enhances PIN plasma membrane allocation, this tallying mechanism can generate both up-the-gradient and with-the-flux type patterns. Since the molecular identity and the location—intracellular, extracellular, or transmembrane—of the auxin concentration and/or flux sensor is unclear, determining the likelihood of these various proposed sensing mechanisms remains challenging.

One aspect common to all these models—implicit or explicit—is the assumption that feedback of auxin on PIN polarity patterns occurs through auxin affecting PIN membrane cycling dynamics. With the finding that auxin is a general inhibitor of endocytosis, and, thereby, also of PIN endocytosis (Paciorek et al., 2005), auxin-dependent PIN polarization appeared to have received its experimental support. Assuming a feedback of extracellular, apoplastic auxin levels on PIN endocytosis, this effect would give rise to the type of positive feedback necessary for self-organized patterning (Figure 1D). Following these findings, “up-the-gradient,” phyllotaxis type PIN polarization could arise from auxin levels of neighboring cells via their surrounding apoplast, reducing PIN endocytosis, while “with-the-flux patterning” could arise if, for example, an increase in external auxin levels could be read-out as an increased (ef)flux, resulting via reduced PIN endocytosis in a further increase in efflux.

Importantly, the results of Paciorek et al. (2005) heavily relied on the use of Brefeldin A (BFA), an inhibitor of endosome-to-plasma membrane trafficking, with observations of auxin-dependent decreased accumulation of cargos in BFA bodies interpreted as inhibited endocytosis. Recent studies by Jásik et al. (2016) using photoconvertible Dendra2 tagging of PIN2, and Paponov et al. (2019) further investigating the effects of the natural auxin indole-3-acetic acid on BFA-induced internalization raised substantial doubts on this interpretation, reporting no discernable effects of auxin on PIN2 endocytosis. The final blow to the interpretation that auxin inhibits general endocytosis, and thereby PIN internalization, comes from the group originally reporting this effect. Narasimhan et al. (2021) clearly demonstrated no measurable effect of auxin on general endocytosis rates by using Total Internal Reflection Fluorescence microscopy to directly observe individual clathrin-mediated endocytotic events. While the impact of this study on the field of plant cell biology is being discussed in an accompanying Commentary by Schwechheimer et al., this commentary deals with the consequences for models of self-organized auxin transport.

So what then is left of the idea that self-organized patterning may occur through feedback between auxin and PIN polarity? While auxin may not affect PIN polarity patterns by inhibiting membrane endocytosis rates, Jásik et al. (2016) reported that auxin reduces the rate at which newly synthesized PIN proteins arrive at the plasma membrane, and Narasimhan et al. (2021) demonstrate that auxin promotes PIN2 but not PIN1 endocytosis. This leaves the possibility that auxin impacts the polarity of at least certain PIN types, but through a different mechanism than previously thought (Figure 1E). Additional research will be needed to investigate the mechanistic basis of the observed effects as well as whether their impact on PIN membrane patterns is sufficient to support self-organized patterning, and which feedback mechanisms apply for the different PIN types.

In the search for experimental support for self-organized, reciprocal patterning of auxin and PINs it is important to realize what models are: simplifications of reality that are powerful tools for explaining complex phenomena. Thus, when searching for validation they should not be taken too literal. Indeed, a wealth of experimental data suggest that plants may “implement” “up-the-gradient” and “with-the-flux” type patterning not merely through auxin affecting PIN membrane cycling dynamics. Instead, plants may use a substantially more complex molecular machinery, containing transcription factors (e.g. MONOPTEROS; Bhatia et al., 2006), phosphorylation, and dephosphorylation activities (e.g. PINOID, D6 PROTEIN KINASE, and SERINE/THREONINE PROTEIN PHOSPHATASE 2A; Barbosa et al., 2018 ), membrane and cell wall composition, as well as cytoskeletal organization (Li et al., 2021), that we are far from truly understanding. Furthermore, the evidence for auxin-driven PIN polarization as the key determinant of phyllotaxis and venation patterning should be critically reappraised. Various studies suggest that, while PIN polarization and downstream auxin transport significantly enhance and regularize phyllotaxis and venation patterning, PINs appear to be less essential for these patterning processes than often assumed (Guenot et al., 2012; Ravichandran et al., 2020).

## Notes

K.H.T. conceived and wrote the manuscript.

## References

[kiab118-B1] Alim K, Frey E (2010) Quantitative predictions on auxin-induced polar distribution of PIN proteins during vein formation in leaves. Eur Phys J E 33: 165–173 20571847 10.1140/epje/i2010-10604-5

[kiab118-B2] Band LR, Wells DM, Fozard JA, Ghetiu T, French AP, Pound MP, Wilson MH, Yu L, Li W, Hijazi HI, et al (2014) Systems analysis of auxin transport in the Arabidopsis root apex. Plant Cell 26: 862–875 24632533 10.1105/tpc.113.119495 PMC4001398

[kiab118-B3] Barbosa ICR, Hammes UZ, Schwechheimer C (2018) Activation and polarity control of PIN-FORMED Auxin transporters by phosphorylation. Trends Plant Sci 23: 523–538 29678589 10.1016/j.tplants.2018.03.009

[kiab118-B4] Bhatia N, Bozorg B, Larsson A, Ohno C, Jonsson H, Heisler MG (2006) Auxin acts through MONOPTEROS to regulate plant cell polarity and pattern phyllotaxis. Curr Biol 26: 3202–3208 10.1016/j.cub.2016.09.044 PMC5154752 27818174

[kiab118-B5] Cieslak M, Runions A, Prusinkiewicz P (2015) Auxin-driven patterning with unidirectional fluxes. J Exp Bot 66: 5083–5102 26116915 10.1093/jxb/erv262 PMC4513925

[kiab118-B6] De Smet I, Tetsumura T, De Rybel B, Frei dit Frey N, Laplaze L, Casimiro I, Swarup R, Naudts M, Vanneste S, Audenaert D, et al (2007) Auxin-dependent regulation of lateral root positioning in the basal meristem of Arabidopsis. Development 134: 681–690 17215297 10.1242/dev.02753

[kiab118-B7] Di Mambro R, De Ruvo M, Pacifici E, Salvi E, Sozzani R, Benfey PN, Busch W, Novak O, Ljung K, Di Paola L, et al (2017) Auxin minimum triggers the developmental switch from cell division to cell differentiation in the Arabidopsis root. Proc Natl Acad Sci USA 114: E7641–E7649 28831001 10.1073/pnas.1705833114 PMC5594665

[kiab118-B8] Du Y, Scheres B (2018) Lateral root formation and the multiple roles of auxin. J Exp Bot 69: 155–167 28992266 10.1093/jxb/erx223

[kiab118-B9] Feugier FG, Mochizuki A, Iwasa Y (2005) Self-organization of the vascular system in plant leaves: inter-dependent dynamics of auxin flux and carrier proteins. J Theor Biol 236: 366–375 15899502 10.1016/j.jtbi.2005.03.017

[kiab118-B10] Fujita H, Mochizuki A (2006) Pattern formation of leaf veins by the positive feedback regulation between auxin flow and auxin efflux carrier. J Theor Biol 241: 541–551 16510156 10.1016/j.jtbi.2005.12.016

[kiab118-B11] Galvan-Ampudia GS, Julkowska MM, Darwish E, Gandullo J, Korver R, Brunoud G, Haring MA, Munnik T, Vernoux T, Testerink C (2013) Halotropism is a response of plant roots to avoid a saline environment. Curr Biol 23: 2044–2050 24094855 10.1016/j.cub.2013.08.042

[kiab118-B12] Guenot B, Bauer E, Kierzkowski D, Smith RS, Mandel T, Zadnikova P, Benkova E, Kuhlemeier C (2012) Pin1-independent leaf initiation in Arabidopsis. Plant Physiol 159: 1501–1510 22723086 10.1104/pp.112.200402 PMC3425194

[kiab118-B13] Heisler MG, Hamant O, Krupinski P, Uyttewaal M, Ohno C, Jönsson H, Traas J, Meyerowitz EM (2010) Alignment between PIN1 polarity and microtubule orientation in the shoot apical meristem reveals a tight coupling between morphogenesis and auxin transport. PLoS Biol 8: e1000516 20976043 10.1371/journal.pbio.1000516 PMC2957402

[kiab118-B14] Jásik J, Bokor B, Stuchlík S, Mičieta K, Turňa J, Schmelzer E. (2016) Effects of Auxins on PIN-FORMED2 (PIN2) dynamics are not mediated by inhibiting PIN2 endocytosis. Plant Physiol 172: 1019–1031 27506239 10.1104/pp.16.00563 PMC5047079

[kiab118-B15] Jönsson H, Heisler MG, Shapiro BE, Meyerowitz EM, Mjolsness E (2006) An auxin-driven polarized transport model for phyllotaxis. Proc Nat Acad Sci USA 103: 1633–1638 16415160 10.1073/pnas.0509839103 PMC1326488

[kiab118-B16] Li H, von Wangenheim D, Zhang X, Tan S, Darwish-Miranda N, Naramoto S, Wabnik K, De Rycke R, Kaufmann WA, Gutl D, et al. (2021) Cellular requirements for PIN polar cargo clustering in *Arabidopsis thaliana*. New Phytol 229: 351–369 32810889 10.1111/nph.16887 PMC7984064

[kiab118-B17] Ljung K, Hull AK, Celenza J, Yamada M, Estelle M, Normanly J, Sandberg G (2005) Sites and regulation of auxin biosynthesis in Arabidopsis roots. Plant Cell 17: 1090–1104 15772288 10.1105/tpc.104.029272 PMC1087988

[kiab118-B18] Mitchison GJ (1980) A model for vein formation in higher plants. Proc R Soc Lond Biol Sci 207: 79–109

[kiab118-B19] Narasimhan M, Gallei M, Tan S, Johnson A, Vertraeten I, Li L, Rodriguez L, Han H, Himschoot E, Wang R, et al. (2021) Systematic analysis of specific and nonspecific auxin effects on endocytosis and trafficking. Plant Physiol 186: 1122–1142 33734402 10.1093/plphys/kiab134 PMC8195513

[kiab118-B20] Newell AC, Shipman PD, Sun Z (2007) Phyllotaxis: cooperation and competition between mechanical and biochemical processes. J Theor Biol 251: 421–439 18207165 10.1016/j.jtbi.2007.11.036

[kiab118-B21] Paciorek T, Zazímalová E, Ruthardt N, Petrásek J, Stierhof YD, Kleine-Vehn J, Morris DA, Emans N, Jürgens G, Geldner N, et al. (2005) Auxin inhibits endocytosis and promotes its own efflux from cells. Nature 435: 1251–1256 15988527 10.1038/nature03633

[kiab118-B22] Paponov IA, Friz T, Budnyk V, Teale W, Wüst F, Paponov M, Al-Babili S, Palme K (2019) Natural Auxin does not inhibit Brefeldin A induced PIN1 and PIN2 internalization in root cells. Front Plant Sci 10: 574 31143194 10.3389/fpls.2019.00574 PMC6521567

[kiab118-B23] Paponov IA, Teale WD, Trebar M, Blilou I, Palme K (2005) The PIN auxin efflux facilitators: evolutionary and functional perspectives. Trends Plant Sci 10: 170–177 15817418 10.1016/j.tplants.2005.02.009

[kiab118-B24] Rakusova H, Fendrych M, Friml J (2015) Intracellular trafficking and PIN-mediated cell polarity during tropic responses in plants. Curr Opin Plant Biol 23: 116–123 25553419 10.1016/j.pbi.2014.12.002

[kiab118-B25] Ravichandran SJ, Linh NM, Scarpella E, (2020) The canalization hypothesis – challenges and alternatives. New Phytol 227: 1051–1059 32285457 10.1111/nph.16605

[kiab118-B26] Sachs T (1969) Polarity and the induction of organized vascular tissues. Ann Bot 33: 263–275

[kiab118-B27] Smith RS, Guyomarc'h S, Mandel T, Reinhardt D, Kuhlemeier C, Prusinkiewicz P (2006) A plausible model of phyllotaxis. Proc Nat Acad Sci USA 103: 1301–1306 16432192 10.1073/pnas.0510457103 PMC1345713

[kiab118-B28] Stoma S, Lucas M, Chopard J, Schaedel M, Traas J, Godin C (2008) Flux-based transport enhancement as a plausible unifying mechanism for auxin transport in meristem development. PLoS Comp Biol 4: e1000207 10.1371/journal.pcbi.1000207 PMC2565506 18974825

[kiab118-B29] Van Berkel K, de Boer RJ, Scheres B, ten Tusscher KH (2013) Polar auxin transport: models and mechanisms. Development 14: 2253–2268 10.1242/dev.079111 23674599

[kiab118-B30] Wabnik K, Kleine-Vehn J, Balla J, Sauer M, Naramoto S, Reinöhl V, Merks RM, Govaerts W, Friml J (2010) Emergence of tissue polarization from synergy of intracellular and extracellular auxin signaling. Mol Syst Biol 6: 447 21179019 10.1038/msb.2010.103 PMC3018162

